# Individualized Deltoid Landmark and Needle Length for Safe Intramuscular Vaccination in Southeast Asian Adults: An Ultrasound Study

**DOI:** 10.3390/life16050724

**Published:** 2026-04-24

**Authors:** Siwaluk Srikrajang, Narucha Komolsuradej, Pramot Tanutit, Teeranan Laohawiriyakamol, Pattira Boonsri, Chaiwat Chuaychoosakoon

**Affiliations:** 1 Department of Physical Therapy, Faculty of Medicine, Prince of Songkla University, Hat Yai 90110, Thailand; siwaluk.pt@gmail.com; 2 Department of Family and Preventive Medicine, Faculty of Medicine, Prince of Songkla University, Hat Yai 90110, Thailand; narucha.ko@psu.ac.th; 3 Department of Radiology, Faculty of Medicine, Prince of Songkla University, Hat Yai 90110, Thailand; ptanutit@yahoo.com (P.T.); iteerana@medicine.psu.ac.th (T.L.); bpattira@medicine.psu.ac.th (P.B.); 4 Department of Orthopedics, Faculty of Medicine, Prince of Songkla University, Hat Yai 90110, Thailand

**Keywords:** intramuscular vaccination, deltoid muscle, axillary nerve, ultrasound, Southeast Asian adults

## Abstract

**Background/Objectives:** An incorrect intradeltoid injection technique can cause shoulder injury related to vaccine administration, including bursitis, septic arthritis, and axillary nerve injury, particularly when Western-derived landmarks and needle-length tables are applied to smaller-framed Southeast Asian adults. We aimed to define an individualized deltoid injection landmark and needle length that avoid the axillary nerve while ensuring reliable intramuscular delivery in Southeast Asian adults. **Methods:** In this cross-sectional ultrasound study of adults aged ≥18 years, four vertical landmarks below the acromion (individual contralateral 2-fingerbreadth (FB), individual contralateral 3-FB, average 2-FB, average 3-FB) were assessed in two arm positions (adduction and approximately 30° abduction with the hand on the waist). For each combination, we recorded the presence of the axillary nerve and measured skin-to-subcutaneous and deltoid muscle thickness to estimate whether 0.5-, 1-, or 1.5-inch needles would terminate within muscle or penetrate the subdeltoid bursa. **Results:** Eighty-two participants (39 males, 43 females) were included. The axillary nerve was not visualized at the individualized contralateral 2-FB landmark in adduction but was present at 31.7–50.0% of other landmark–position combinations. At the individualized 2-FB site in adduction, mean skin-to-subcutaneous thickness was <12.7 mm and mean skin-to-subdeltoid fascia distance exceeded 12.7 mm in all strata, implying that a 0.5-inch needle would consistently terminate within the deltoid muscle. **Conclusions:** In Southeast Asian adults, the contralateral individualized 2-FB landmark in arm adduction provides a neurovascularly safe window for intradeltoid vaccination, and a 0.5-inch needle offers reliable intramuscular delivery while minimizing the risk of bursal penetration.

## 1. Introduction

Intramuscular vaccination into the deltoid muscle is widely recommended because this site provides adequate vascularity for rapid antigen uptake, sufficient muscle bulk in most adults, and convenient access for large-scale immunization programs [1,2]. However, an incorrect intradeltoid technique can result in shoulder injury related to vaccine administration (SIRVA), bursitis, synovitis, or axillary nerve injury, which can cause severe shoulder pain, functional limitation, and prolonged morbidity that is disproportionate to expected post-injection discomfort, and in some cases requires surgical intervention such as arthroscopic debridement or capsular release [3,4,5,6,7].

Most of the existing guidelines, including the Centers for Disease Control and Prevention (CDC) guidelines, were developed using anthropometric data and clinical experience from Western populations and typically recommend fixed landmarks (for example, 2–3 fingerbreadths (FB) or 3–5 cm below the acromion) and standard needle lengths [8,9,10,11]. Emerging anatomical and imaging evidence in Asian cohorts, including Southeast Asian populations, suggests that these “average” parameters may not be directly applicable [12]. On average, Southeast Asians are shorter and lighter and exhibit different patterns of deltoid muscle thickness, subcutaneous fat distribution, and shoulder morphology compared with Western populations. Consequently, Western-derived average landmarks and needle lengths may lead to excessive penetration beyond the deltoid muscle into the subdeltoid bursa or joint capsule, thereby increasing the risk of SIRVA, or to injections placed too inferiorly in relation to the course of the axillary nerve.

In routine practice, deltoid landmarking is often based on a presumed “average” distance below the acromion, typically described in FB or as a fixed linear measurement. This approach neglects inter-individual variation in shoulder size, hand size, and deltoid morphology, which may be particularly pronounced in Southeast Asian populations with a wide range of body habitus, from underweight to obese. As a result, a fixed landmark may position the injection too proximally, overlapping the subacromial–subdeltoid bursa, or too distally, approaching the region where the axillary nerve winds around the surgical neck of the humerus. Both scenarios theoretically increase the risk of intra-articular or intraneural injection.

These observations suggested that a more individualized strategy for intradeltoid vaccination might be preferable for Southeast Asian patients. Instead of relying on a single average landmark, the injection site could be located using each patient’s own FB measured from the acromion, thereby scaling the target zone to that person’s shoulder dimensions. In theory, such an individualized FB distance would better position the needle within a mid-deltoid “safe window,” keeping the tip away from the subacromial–subdeltoid bursa superiorly and from the typical course of the axillary nerve inferiorly, while improving the consistency of intramuscular delivery in a heterogeneous population. At the same time, a truly optimized technique would also require accurate estimation of the penetration depth needed to reach the deltoid muscle without excessive advancement beyond it.

The thickness of the skin, subcutaneous tissue, and deltoid muscle varies substantially between individuals according to sex, body mass index (BMI), and local fat distribution [13,14]. Systematic evaluation of these tissue layers in a Southeast Asian population allows determination of the minimal depth required to traverse the skin–subcutaneous envelope and achieve reliable intramuscular deposition, while avoiding entry into deeper structures.

The primary objective of this study was to identify an appropriate intradeltoid injection landmark by comparing a conventional average FB distance from the acromion with an individualized contralateral FB measurement, with the aim of avoiding axillary nerve injury. In addition, this study sought to determine the thickness of the skin, subcutaneous tissue, and deltoid muscle at the proposed injection site, in order to recommend a suitable needle length that reliably delivers the injectate into the muscle. Ultrasound was used to measure tissue thickness, and these measurements were integrated with the nominal needle length to estimate the proportion of the needle that should be inserted to achieve safe and effective intramuscular injection.

## 2. Materials and Methods

This cross-sectional ultrasound study was conducted at the Faculty of Medicine, Prince of Songkla university, Thailand. The study was approved by the institutional ethics committee of our university (approval number: 67-088-30-2). Adult Thai participants, representing a Southeast Asian population, were recruited according to predefined inclusion and exclusion criteria. Eligible participants were Thai adults aged ≥18 years who received care at the hospital, primarily within the outpatient department, and who volunteered to taken part in the study. Individuals were excluded if they had a history of fracture involving the shoulder girdle (scapula, clavicle, or proximal humerus) or any central or peripheral neurological disorder affecting deltoid muscle function. All participants provided written informed consent prior to enrollment. The target sample size was determined using a single-group mean formula, assuming a two-sided confidence level of 95% $\left(Z_{\alpha /2}|1.96\right)$, an expected standard deviation of 0.75 based on Nakajima et al. (2017) [11], and an acceptable absolute error of 0.2 (2%); in practice, 83 participants were enrolled: 39 in the 18–60-year-old group and 44 in the >60-year-old group.

To explore potential age-related differences in deltoid morphology, participants were stratified into two age groups: 18–60 years and >60 years. After consent was obtained, basic demographic and clinical data were collected by interview, including sex, age, weight, height, occupation, smoking and alcohol consumption history, comorbidities, and hand dominance. Ultrasound examinations of both shoulders were then performed to evaluate the deltoid region. All scans were conducted by a single experienced investigator with expertise in musculoskeletal ultrasonography, using a high-frequency linear transducer. The primary landmarks were defined using FB measurements from the lateral edge of the acromion process. For every participant, the widths of 2-FB and 3-FB were measured on the index, middle, and ring fingers of the contralateral hand, and these individual values were recorded. The contralateral hand was specifically used for these measurements to avoid potential interference with the injection site and to better reflect real-world vaccination practice, in which healthcare providers typically use the opposite hand to identify anatomical landmarks. In addition, average population-based 2-FB and 3-FB distances were defined from prior pilot data and used as “average” landmarks [12].

**Figure 1:**
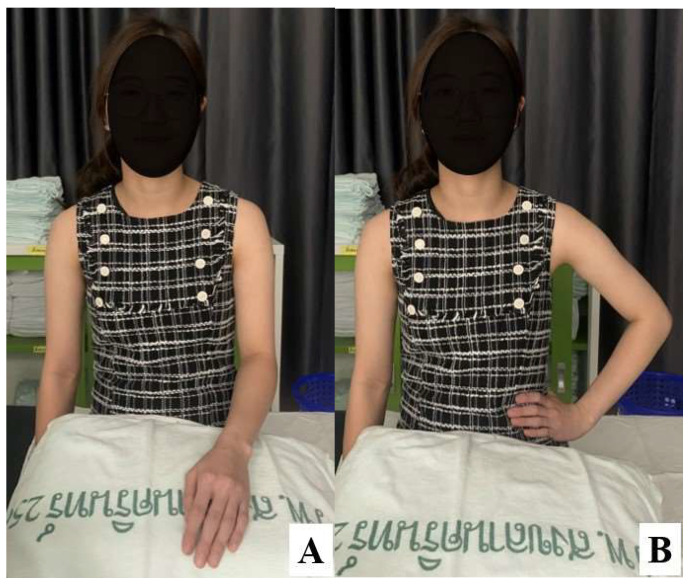
Arm positions during landmark assessment: (**A**) arm resting alongside the trunk; (**B**) arm abducted to approximately 30° with the hand placed on the waist.

Four landmark conditions were therefore evaluated on each arm: (1) individual contralateral 2-FB below the acromion, (2) individual contralateral 3-FB below the acromion, (3) average 2-FB below the acromion, and (4) average 3-FB below the acromion. For the individualized landmarks, the distal interphalangeal (DIP) joint of the index finger was positioned at the lower border of the mid-acromion, and the combined widths of the index and middle fingers (2-FB) or the index, middle, and ring fingers (3-FB) of the contralateral hand were used to define the vertical distance below the acromion. For each landmark condition, two clinically relevant arm positions were assessed: (a) the arm resting alongside the trunk (Figure 1A) and (b) the arm abducted to approximately 30° with the hand placed on the waist (Figure 1B). At every combination of landmark (four) and arm position (two), the ultrasound probe was placed transversely over the deltoid to visualize the skin, subcutaneous tissue, deltoid muscle, humeral cortex, and the axillary nerve. To minimize false-negative nerve detection related to ultrasound’s finite spatial resolution or a deep nerve course, scanning parameters were standardized so that the humeral cortex was always clearly visualized; depth, gain, and focal zone were adjusted to ensure that the entire subdeltoid compartment lay within the diagnostic window. In addition, we did not rely on a single static view. When the axillary nerve was not immediately apparent at the landmark of interest, a dynamic scanning protocol was used, translating the probe in superior–inferior and anterior–posterior directions, and, if necessary, the nerve was first identified in the quadrangular space and then traced anteriorly along the surgical neck. Using this systematic, landmark-based approach, we were able to visualize the axillary nerve in all participants while using a high-frequency linear transducer (2.9–9.9 MHz) that provided sufficient penetration and axial resolution to distinguish the nerve from the surrounding deltoid fibers and posterior humeral circumflex artery. Using this approach, the axillary nerve was identified in all participants (100%). This success rate aligns with the recent literature [15], suggesting that high-resolution ultrasound is highly reliable for axillary nerve visualization.

**Figure 2:**
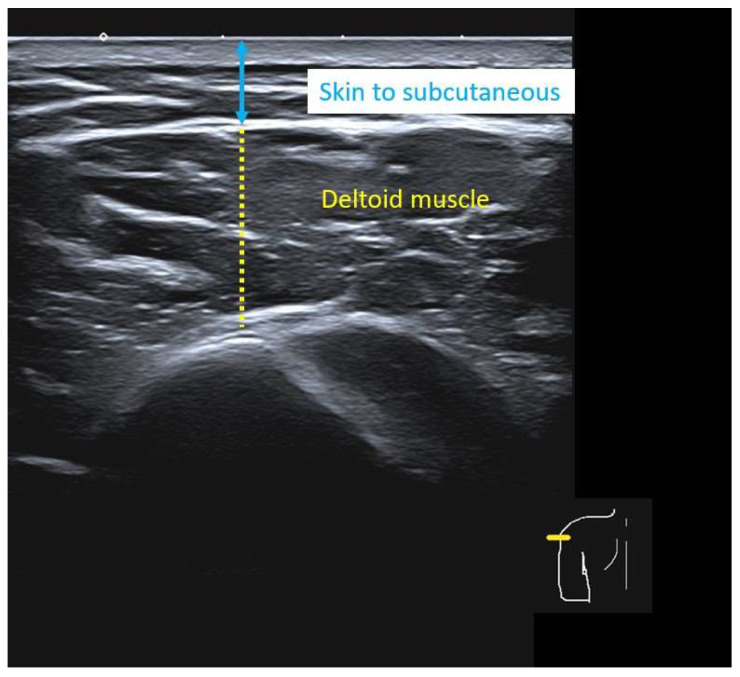
Ultrasonographic image showing the thickness from the skin surface to the deep border of the subcutaneous fat pad (blue double arrowhead) and the deltoid muscle thickness from this border to the subdeltoid fascia (yellow dotted line).

**Figure 3:**
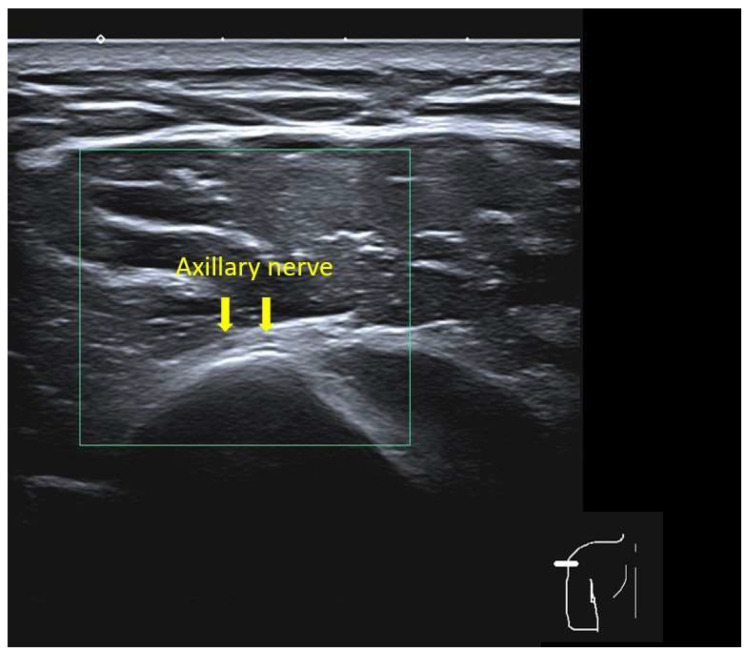
Ultrasonographic image demonstrating the axillary nerve (yellow arrow).

Using frozen ultrasound images, the thickness of the skin, subcutaneous tissue, and deltoid muscle was measured perpendicular to the skin surface at each landmark and arm position. The thickness of each tissue layer was recorded, including the distance from the skin surface to the deep border of the subcutaneous fat pad and the thickness of the deltoid muscle from this border to the subdeltoid fascia (Figure 2). The location of the axillary nerve relative to each landmark was identified whenever visible, based on its characteristic hyperechoic fascicular appearance deep to the deltoid and near the surgical neck of the humerus (Figure 3). Any landmark at which the axillary nerve was visualized was classified as unsafe because of the potential risk of iatrogenic axillary nerve injury.

From these measurements, the vertical distance between each landmark and the axillary nerve was calculated to determine which landmark–position combinations provided the greatest safety margin and could therefore be considered “safest” for intradeltoid injection with respect to avoiding axillary nerve injury. In addition, the measured thicknesses of the skin, subcutaneous tissue, and deltoid muscle were integrated with standard vaccine needle lengths to select a needle length that achieves reliable intramuscular delivery while avoiding both underpenetration into subcutaneous tissue and overpenetration beyond the muscle layer. These data were used to propose age-appropriate and habitus-adjusted recommendations for both injection landmark (individual versus average FB-based) and needle length for intradeltoid vaccination in Thai adults.

Descriptive statistics were reported as means ± standard deviations (SDs) and as incidence ratios indicating intradeltoid penetration at each needle depth. Statistical analyses were performed using R with the epicalc package (version 3.4.3, R Foundation for Statistical Computing, Vienna, Austria). Independent *t*-tests were applied to compare mean age, height, weight, BMI, and skin–subcutaneous and deltoid muscle thicknesses. Incidence ratios of intradeltoid penetration across needle depths were compared using chi-square tests of independence or Wilcoxon rank-sum tests, as appropriate. Pearson correlation coefficients were calculated to examine relationships between skin–subcutaneous and deltoid muscle thicknesses and participants’ weight, height, and BMI in men and women. A *p* value < 0.05 was considered statistically significant.

## 3. Results

Thirty-nine males and forty-three females were included and categorized into two age groups: 39 participants aged 18–60 years and 44 participants older than 60 years. In the 18–60-year group, the mean age was 41.5 ± 13.1 years in males and 37.4 ± 12.3 years in females, the mean height was 170.4 ± 6.9 cm in males and 157.8 ± 5.7 cm in females, and the mean weight was 67.7 ± 8.7 kg in males and 58.9 ± 13.8 kg in females. In participants older than 60 years, the mean age was 69.1 ± 6.1 years in males and 68.6 ± 4.9 years in females, the mean height was 166.4 ± 6.5 cm in males and 154.6 ± 4.4 cm in females, and the mean weight was 65.6 ± 11.7 kg in males and 59.0 ± 11.1 kg in females.

The axillary nerve was present at the contralateral individualized 2-FB landmark in arm adduction in 0% of cases. At the other adducted landmarks—contralateral individualized 3-FB, average 2-FB, and average 3-FB—the axillary nerve was identified in 31.7%, 1.2%, and 35.4% of shoulders, respectively. In the abducted position with internal rotation, the corresponding percentages for contralateral individualized 2-FB, contralateral individualized 3-FB, average 2-FB, and average 3-FB were 1.2%, 50%, 6.1%, and 35.4%, respectively.

**Figure 4:**
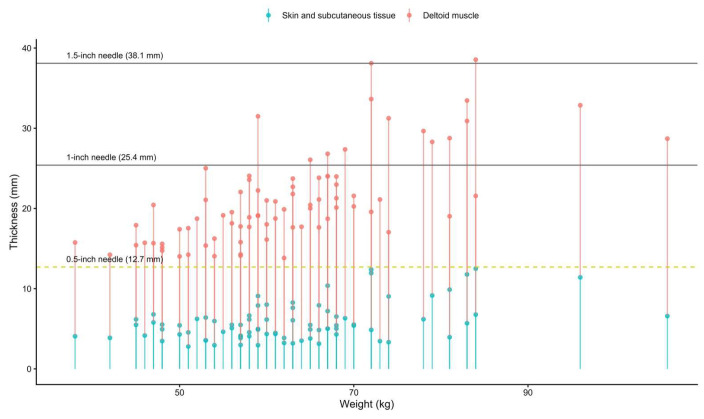
Individual skin–subcutaneous and deltoid muscle thicknesses at the contralateral individualized 2-FB landmark in arm adduction plotted against body weight for each shoulder (83 participants, 166 shoulders). Each vertical pair of points represents, for a single participant, the thickness of the skin and subcutaneous tissue (blue) and the total deltoid muscle thickness (red). The horizontal dotted yellow line indicates the nominal needle length of 0.5 inch (12.7 mm).

At the contralateral individualized 2-FB landmark in arm adduction, the mean skin-to-subcutaneous thickness in participants aged 18–60 years and those older than 60 years was 4.7 ± 1.2 mm in males and 7.3 ± 3.4 mm in females, and 4.9 ± 1.7 mm in males and 6.0 ± 2.3 mm in females, respectively. The corresponding deltoid muscle thicknesses were 16.6 ± 3.4 mm in males and 14.9 ± 5.6 mm in females aged 18–60 years, and 15.6 ± 3.3 mm in males and 14.7 ± 3.2 mm in females older than 60 years. Simulated vaccine administration using different needle lengths (Figure 4) showed that with a 0.5-inch needle (12.7 mm), the needle tip terminated within the deltoid muscle without reaching the subdeltoid fascia in 100% of cases. With a 1-inch needle (25.4 mm), 81.7% of simulations demonstrated penetration beyond the deltoid muscle into the subdeltoid bursa, whereas with a 1.5-inch needle (38.1 mm), 97.56% demonstrated penetration beyond the subdeltoid fascia.

### Correlation Analysis by Sex

**Figure 5:**
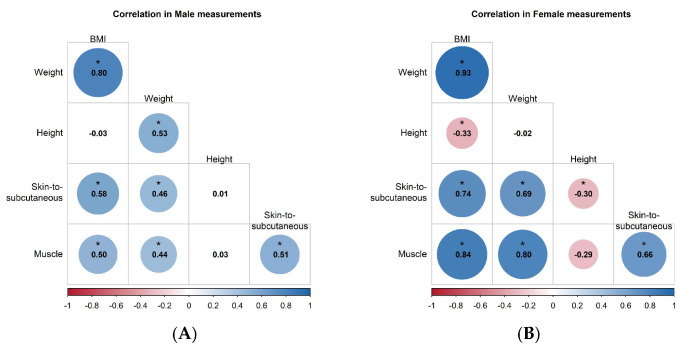
Correlation matrices of anthropometric and deltoid tissue-thickness measures in males (**A**) and females (**B**); circle size and color indicate the magnitude and direction of Pearson correlations, with asterisks denoting *p* < 0.05.

In males, weight showed a strong positive correlation with BMI (r = 0.80) and moderate positive correlations with height (r = 0.53), skin-to-subcutaneous thickness (r = 0.58), and muscle thickness (r = 0.50). Height had essentially no correlation with skin-to-subcutaneous thickness (r = 0.01) or muscle thickness (r = 0.03). Skin-to-subcutaneous and muscle thickness were moderately positively correlated (r = 0.51), indicating that thicker subcutaneous fat tended to accompany thicker deltoid muscle (Figure 5A). In females, weight had an even stronger positive correlation with BMI (r = 0.93) and strong positive correlations with skin-to-subcutaneous thickness (r = 0.74) and muscle thickness (r = 0.80), but almost no correlation with height (r = −0.02). Height showed modest negative correlations with skin-to-subcutaneous thickness (r = −0.30) and muscle thickness (r = −0.29), suggesting that shorter women tended to have thicker superficial fat and muscle. Skin-to-subcutaneous and muscle thickness were strongly positively correlated (r = 0.66), again indicating that greater superficial fat was associated with greater deltoid muscle thickness in women (Figure 5B).

## 4. Discussion

Using the individualized contralateral 2-FB landmark in the arm-adduction position, together with a 0.5-inch needle penetration for intradeltoid vaccination, helps to avoid the axillary nerve and ensures that the needle tip terminates within the deltoid muscle.

When either component is chosen incorrectly, clinically important complications can occur. An injection given too low on the deltoid or too close to the course of the axillary nerve risks direct needle trauma or chemical neuritis, leading to deltoid weakness, sensory disturbance over the lateral shoulder, and prolonged functional limitation. Conversely, inappropriate needle penetration depth can result in subcutaneous deposition, with reduced immunogenicity, or overpenetration into the subacromial–subdeltoid bursa or joint capsule, causing SIRVA presentations such as bursitis, adhesive capsulitis, or even septic arthritis and sometimes necessitating surgical intervention.

From a neuroanatomical perspective, the absence of the axillary nerve at this landmark is clinically important. Cadaveric studies consistently locate the main trunk and anterior branch of the axillary nerve around 5 cm below the acromion, with substantial inter-individual variability in both the vertical and horizontal planes. Clinical case reports of vaccination-related axillary neuropathy typically describe injections given at or below this level, often with standard needle lengths of 1–1.5 inches. These injuries may present with acute shoulder pain, subsequent deltoid atrophy, loss of abduction strength, and sensory changes over the regimental badge area, and recovery often requires many months of rehabilitation. By contrast, our ultrasound mapping indicates that the individualized 2-FB landmark lies superior to the region where the nerve is most frequently encountered. Selecting a site where the nerve is consistently absent rather than merely “on average distant” provides a buffer against real-world deviations from ideal technique, such as minor errors in palpation, slight inferior drift of the needle, or inadvertent deepening of the trajectory when the injector stabilizes the syringe with firm pressure on the skin. We agree that classifying a landmark as “unsafe” whenever the axillary nerve is visualized represents a conservative operational definition, and we recognize the limitations of “non-visualization.” Using a systematic high-resolution ultrasound protocol with standardized scanning planes, dynamic transverse sweeps at each landmark–position combination, and a predefined strategy when the nerve was not initially visible, we achieved a 100% axillary nerve visualization rate in our cohort, consistent with prior work demonstrating reliable identification of the axillary nerve in healthy volunteers. Nevertheless, a finding of “0% visualized within the injection corridor” at the individualized 2-FB site does not prove absolute anatomical absence, but rather indicates that no nerve was detectable under our protocol and should be interpreted as a strong, but not absolute, safety indicator.

Equally important is the question of depth. In practice, vaccinators must navigate a narrow therapeutic window: the needle must pass through skin and subcutaneous fat to reach the muscle, but it should not continue into the subdeltoid bursa or joint capsule. Our measurements at the contralateral individualized 2-FB site showed that the thickness of the skin and subcutaneous layer was consistently less than 12.7 mm, and that the total distance from the skin to the subdeltoid fascia was greater than 12.7 mm in all participants, leaving a residual muscle layer beyond the needle tip. These findings imply that full advancement of a 0.5-inch needle, perpendicular to the skin, reliably places the needle tip within the deltoid muscle while maintaining a margin from the deep fascia. This conclusion is concordant with our prior MRI simulation, which showed 100% intramuscular positioning of a 0.5-inch needle at a 2-FB landmark without penetration of the subdeltoid bursa [13]. Unlike MRI, which was performed in a supine position and could only model static needle paths, the present ultrasound study reflects the actual anatomical relationships during intradeltoid vaccination in the seated position and, importantly, confirms in real time that the axillary nerve is not present within the injection trajectory at this individualized landmark.

In addition, the correlation analysis offers insight into how body habitus influences local tissue layers at the injection site. In males, both skin-to-subcutaneous and deltoid muscle thickness showed only moderate positive correlations with weight and almost no association with height, suggesting that increases in body size do not translate into large or predictable changes in soft-tissue depth at the individualized 2-FB landmark. In females, these relationships were stronger, with skin-to-subcutaneous and muscle thickness demonstrating high positive correlations with weight and modest negative correlations with height, indicating that heavier but shorter women may have proportionally thicker superficial fat and muscle over the deltoid. Importantly, in both sexes, skin-to-subcutaneous and muscle thickness were positively correlated, meaning that individuals with more superficial fat tended also to have thicker deltoid muscle rather than a simple trade-off between fat and muscle. These patterns help explain why a uniform 0.5-inch needle remained adequate across a wide range of body sizes in our cohort: although tissue thickness varied with weight, the coupled increase in muscle thickness preserved a safety margin between the needle tip and the subdeltoid fascia at the individualized 2-FB site.

The clinical relevance of this combined landmark-and-depth strategy becomes evident when juxtaposed with reports of shoulder injury related to vaccine administration (SIRVA). Bursal injection and inadvertent intra-articular deposition have been associated with subacromial–subdeltoid bursitis, adhesive capsulitis, and septic arthritis; some patients require arthroscopic debridement after failure of conservative management [16]. At the same time, axillary nerve injury has been reported after injections placed too low in the deltoid or delivered with long needles at conventional “2–3-FB” sites derived from Western anthropometry [17,18,19,20,21]. The traditional emphasis on body weight-based needle-length tables does not account for regional differences in body habitus, shoulder dimensions, or fat distribution, and our previous MRI study demonstrated that rigid application of these tables to Thai adults results in a high simulated rate of bursal penetration with 1-inch and 1.5-inch needles. The present ultrasound study now adds direct neurovascular evidence, showing that inferior sites near 3-FB below the acromion lie closer to the typical course of the axillary nerve, whereas the individualized 2-FB site does not. When our individualized protocol was compared directly with commonly recommended international landmarks, the axillary nerve entered the injection corridor in 1.2% of participants at the average 2-FB site in adduction (6.1% in abduction) and in 35.4% at the average 3-FB site in both positions, while no axillary nerve was visualized within the injection corridor at the contralateral individualized 2-FB site with a 0.5-inch needle (0%).

An additional advantage of using a contralateral individualized 2-FB measurement is that it inherently scales the landmark to the patient’s own hand and shoulder size, rather than relying on a fixed anthropometric average. This approach recognizes that the absolute distance corresponding to “two FB” varies meaningfully between individuals. For smaller adults, applying a fixed distance derived from another population may effectively move the injection closer to the nerve and the bursa; in larger adults, it may position the injection more superficially, increasing the risk of subcutaneous deposition, particularly if longer needles are shortened in practice because the injector does not fully seat the hub on the skin. Using each person’s own finger breadth to determine the vertical position helps to center the injection in the mid-deltoid region across a range of body sizes, while the 0.5-inch needle length standardizes depth in a way that is straightforward to apply in busy vaccination settings.

Although minor bilateral differences in hand and finger dimensions have been reported in healthy adults [22], these asymmetries are small and, in our experience, unlikely to introduce clinically meaningful error when the contralateral hand is used to determine the 2-FB landmark. In this study, the safety of the contralateral individualized 2-FB site was supported directly by ultrasound-based confirmation of the axillary nerve’s absence within the injection corridor and by the compatible skin-to-muscle and total tissue thickness at this landmark. Thus, even if subtle hand-size asymmetry is present, the imaging findings suggest that using the contralateral hand to scale the landmark remains a safe and practical strategy for intradeltoid vaccination.

**Figure 6:**
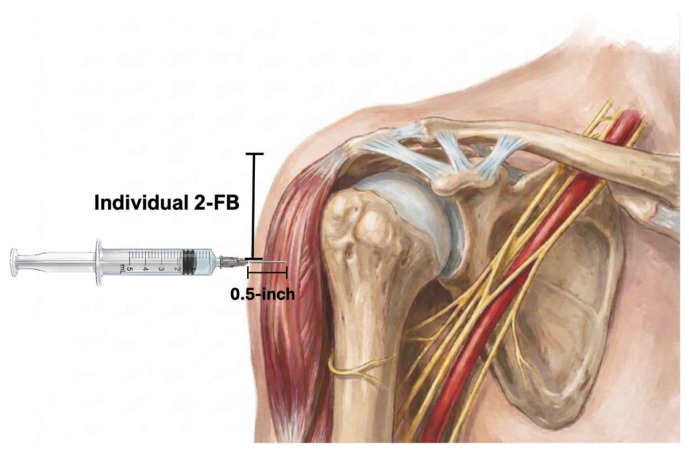
Illustration of the intradeltoid injection site (two fingerbreadths below the acromion) and a 0.5-inch needle advanced perpendicular to the skin into the deltoid muscle.

These findings also have implications for training and guideline development. Current recommendations from international bodies often specify a relatively broad target area (for example, 2–3 FB below the acromion) and then offer tables of needle length according to body weight or BMI. Although such frameworks are useful, they may be too coarse to protect smaller-framed populations from over-penetration or from injections that encroach on the axillary nerve. Our data support adding two refinements for Thai adults (Figure 6): first, specifying the contralateral individualized 2-FB landmark as the preferred injection point; and second, endorsing a 0.5-inch needle as the default length for routine adult intradeltoid vaccination, with longer needles reserved for clearly defined exceptional circumstances. In addition, vaccination staff should be explicitly trained to confirm that the arm remains in a relaxed, adducted position before landmarking and needle insertion, particularly in high-throughput mass vaccination settings. Because this recommendation rests on direct imaging of both tissue thickness and nerve location, it may be more robust than extrapolating from studies conducted in Western cohorts whose anthropometrics differ substantially.

Nevertheless, the present study should be interpreted in light of its limitations. Ultrasound examinations were performed under controlled conditions with participants in a standardized position, whereas real-world vaccination occurs across a spectrum of postures and degrees of muscle relaxation. Although we attempted to account for potential soft-tissue compression by considering the margin between the needle tip and subdeltoid fascia, we did not quantify the additional depth introduced by firm syringe pressure, a mechanical “compression factor” that may occur during real-world practice and influence needle penetration. Our sample did not heavily represent individuals with extreme body habitus; it is possible that in very obese or extremely thin adults, a 0.5-inch needle may be insufficient or, conversely, unnecessarily deep. Finally, while the axillary nerve was not visualized within the injection corridor at the individualized 2-FB landmark, ultrasound has finite spatial resolution, and rare anatomical variants cannot be entirely excluded. We also did not directly assess bilateral hand-size asymmetry in this cohort; although such differences are generally small, the use of contralateral fingerbreadth as a scaling unit may introduce minor landmark variation and should therefore be regarded as a potential limitation of this method. In addition, we did not collect data on arm circumference, which may represent a useful, region-specific anthropometric measure for refining needle-length recommendations, and future studies should incorporate this parameter to further individualize intradeltoid vaccination protocols.

## 5. Conclusions

By demonstrating that the contralateral individualized 2-FB landmark is free of the axillary nerve on ultrasound and that both the superficial and total tissue thicknesses at this site are compatible with a 0.5-inch intramuscular injection in adults with typical body habitus, this study provides a tightly reasoned anatomical basis for a simplified vaccination protocol in Thai adults. Implementing this protocol has the potential to reduce the risk of axillary neuropathy and SIRVA while maintaining efficient, reproducible intradeltoid vaccination. Future work should assess clinical outcomes when these recommendations are applied prospectively in mass vaccination campaigns and should explore whether similar individualized landmarking strategies are needed for other populations with different anthropometric characteristics.

## Data Availability

Data available on request due to restrictions due to ethical reasons.

## Abbreviations

The following abbreviations are used in this manuscript:

IM	intramuscular
SIRVA	shoulder injury related to vaccine administration
FB	fingerbreadth
MRI	magnetic resonance imaging
CDC	Centers for Disease Control and Prevention
BMI	body mass index
SD	standard deviation
cm	centimeter
mm	millimeter
